# Bioprospecting metagenomes: glycosyl hydrolases for converting biomass

**DOI:** 10.1186/1754-6834-2-10

**Published:** 2009-05-18

**Authors:** Luen-Luen Li, Sean R McCorkle, Sebastien Monchy, Safiyh Taghavi, Daniel van der Lelie

**Affiliations:** 1 Biology Department, Brookhaven National Laboratory, Upton, New York 11973, USA; 2 BioEnergy Science Center, Oak Ridge National Laboratory, Oak Ridge, Tennessee 37831, USA

## Abstract

Throughout immeasurable time, microorganisms evolved and accumulated remarkable physiological and functional heterogeneity, and now constitute the major reserve for genetic diversity on earth. Using metagenomics, namely genetic material recovered directly from environmental samples, this biogenetic diversification can be accessed without the need to cultivate cells. Accordingly, microbial communities and their metagenomes, isolated from biotopes with high turnover rates of recalcitrant biomass, such as lignocellulosic plant cell walls, have become a major resource for bioprospecting; furthermore, this material is a major asset in the search for new biocatalytics (enzymes) for various industrial processes, including the production of biofuels from plant feedstocks. However, despite the contributions from metagenomics technologies consequent upon the discovery of novel enzymes, this relatively new enterprise requires major improvements. In this review, we compare function-based metagenome screening and sequence-based metagenome data mining, discussing the advantages and limitations of both methods. We also describe the unusual enzymes discovered via metagenomics approaches, and discuss the future prospects for metagenome technologies.

## Background

In recent years, biofuels have attracted great interest as an alternative, renewable source of energy in the face of the ongoing depletion of fossil fuels, our energy dependence on them, and our growing environmental awareness of the critical consequences of burning such fuels. Plant biomass, the most abundant biopolymer on earth, has long been recognized as a potential sustainable source of mixed sugars for biofuel production. However, breakthrough technologies are still needed to overcome the several barriers to developing cost-effective processes for converting biomass to fuels and chemicals [1]. As yet, we have an incomplete understanding of the plant cell wall and its deconstruction and conversion; considerable research will be needed to better appreciate the fundamental and applied aspects of enzymatic hydrolysis and microbial hydrolysis and/or fermentation of plant cell walls.

Estimates suggest that approximately 4–6 × 10^30 ^prokaryotes inhabit the earth [2]. Being the oldest life form, prokaryotic microorganisms have evolved and accumulated remarkable physiological and functional diversity, thereby constituting the world's major reserve of genetic diversity. Traditional methods to tap this information are by cultivating the microorganisms, subsequently screening individual ones for the requisite phenotypes. However, about 95% to 99.9% of microorganisms have not been cultured by standard laboratory techniques [3]. One way to overcome this limitation is by improving cultivation-based methodologies [4,5].

As a cultivation independent approach, Pace and colleagues [6] proposed a way to isolate directly the collective genomes from all microorganisms in a given habitat, and, in 1991, Schmidt *et al*. [7] undertook the first metagenome-based community characterization on amplified 16S rRNA genes. The subsequent improvement of sequencing technologies made feasible the metagenome shot-gun sequencing of environmental samples; however, most environmental communities are far too complex to be fully sequenced in this manner. Initial attempts were made to reconstruct the metagenomes of viral communities in the ocean and human feces [8-10], and subsequently of samples from the Sargasso Sea [11] and a biofilm from an acid mine drainage (AMD) [12]. However, since most marine communities are far richer in species diversity than the AMD biofilm, on the order of 100 to 200 species per milliliter of water [13,14], this further complicated their sequencing and assembly. Soil communities are even more complex, with an estimated species richness of about 4,000 species per gram of soil [13-15]. On the other hand, with recent developments in high-throughput sequencing technologies, such as the 454 pyrosequencing (GS FLX Titanium Series, 454 Life Science, Roche) partly mitigating this problem, metagenomics is becoming an increasingly sophisticated approach to the study of complex DNA samples directly isolated from defined habitats [16]. According to the Genomes OnLine Database (GOLD) [17] until January 2009, 137 metagenomics projects were in various stages of sequencing, 72% of which were derived from environmental samples, 23% from endobiotic samples, along with 5% synthetic metagenomes. Forty-six of these projects were completed; data are available on the website Integrated Microbial Genomes with Microbiome Samples [18]

Here, we review some recent metagenomic approaches to mining complex microbial communities, comprising both cultivable and non-cultivable microorganisms, for novel biocatalytic enzymes, such as glycosyl hydrolases (GHase) for industrial uses and biofuel production. We also discuss the advantages and limitations of the strategies and tools developed for targeted screening, as well as the future prospects of metagenomics in bioprospecting for new enzymes.

### Strategies for target-gene enrichment

In principle, directly isolating metagenomic DNA from the environment implies unbiased genomic representation; however, biases are introduced during its isolation, for example, resulting from differences in cell lyses. In searching for relatively under-represented genes, enrichment can increase the probability of their cloning, and hasten the process of discovering new genes. By exposing microbial communities to a selective pressure expected to entail the enrichment of microorganisms displaying the desired phenotypes (including substrate utilization, physical-, chemical-, and nutritional-selective conditions), the numbers of those community members with the desired phenotypes and corresponding target genes are successfully boosted. For example, using DNA isolated from enrichment cultures grown on cellulose as their major carbon source increased from three- to four-fold the isolation of GHase with cellulase activity from metagenome libraries, compared with the isolates from libraries made directly from total environmental DNA [19]. Also, we can remove eukaryotic community members by size-selective filtration, leaving behind enriched prokaryotic and archaeal populations [11]. Other enrichment techniques include stable isotope probing, affording a means to isolate microorganisms actively metabolizing the substrate and undergoing replication [20,21], suppressive subtractive hybridization [22-24], differential expression analysis [25], phage display, and affinity capture (reviewed by Cowan *et al*. [26]).

### Strategies for prospecting novel enzymes from metagenomes

Having isolated metagenomic DNA, two complementary approaches can be used for prospecting novel enzymes from it; function-based screening of expression libraries and sequence-based gene searches. In the former, metagenomic expression libraries are constructed and screened for target enzyme activities. For the latter, target genes are cloned after being amplified from metagenomic DNA by using polymerase chain reaction with conserved sequences as primers; alternatively, they may be directly discovered from metagenome sequence databases using bioinformatics tools, subsequently amplified, and cloned in the appropriate expression systems. Below, we detail these two approaches.

#### Metagenome expression libraries (function-based screening)

Metagenome expression libraries are constructed by inserting fragmented metagenomic DNA into expression vectors based on plasmids, cosmids, fosmids, or phages, after which gene expression is examined in a suitable host system. The advantage of directly screening for enzymatic activities from metagenome libraries is that researchers access previously unknown genes and their encoded enzymes. Furthermore, the sequences and enzyme activities are functionally guaranteed. However, some potential drawbacks compromise this approach. Thus, before a clone correctly expresses an active enzyme, several requirements must be met. First, when functional enzymatic activity depends on more than one genetic subunit, the clone must contain the complete gene sequence, or even a gene cluster. This problem can be resolved by selecting suitable vector systems. For small target genes, DNA fragment libraries with inserts between 2 and 10 kilobase (kb) are constructed in plasmids or Lambda expression vectors, and then screened for enzyme expression. Larger gene clusters, preferentially necessitate expression libraries with inserts between 20 and 40 kb in cosmids and fosmids, and up to 100 to 200 kb in bacterial artificial chromosome vectors. Although common *E. coli *host strains have relaxed requirements for promoter recognition and translation initiation, many genes from environmental samples may not be expressed efficiently in heterologous hosts due to differences in codon usage, transcription and/or translation initiation signals, protein-folding elements, post-translational modifications, such as glycosylation, or toxicity of the active enzyme. This obstacle is overcome partly by selecting suitable vector systems containing apposite transcription and translation-initiation sequences, and using suitable expression hosts, such as the *E. coli *Rosetta strains (Novagen, Madison, Wisconsin, USA) that contain the tRNA genes for rare amino acid codons [27], or co-expression of the chaperone proteins, such as GroES, GroEL, and heat-shock proteins [28,29]. Alternatively, host systems such as insect cells, the yeast *Pichia pastoris*, and bacterial hosts such as *Pseudomonas putida*, *Streptomyces lividans*, or *Bacillus subtilis *were suitably improved for heterologous gene expression [30]. Furthermore, several modified function-based methods exist specifically for exploring metagenome libraries. Thus, Uchiyama and colleagues [31] developed substrate-induced gene-expression screening to rapidly identify clones that can be induced by a target substrate and display catabolic gene expression, while metabolite-regulated expression detects clones generating quorum-sensing gene-inducing compounds [32].

Function-based metagenome library screening has uncovered a wide range of biocatalysts. Here, we highlight several published results that screened for polysaccharide and plant cell wall biomass-degrading enzymes, most belonging to GHase families. In most cases, colorimetric-based analyses on agar plates employing dye-linked substrates or reaction products staining were used for preliminary screening. Candidate clones were then confirmed by enzyme activity assays.

Amylases attract much industrial interest and are the focus of many metagenome studies. Richardson *et al*. [33], Voget *et al*. [34], Yun *et al*. [35], and Lämmle *et al*. [36] detailed novel amylolytic enzyme activities from metagenome libraries; some of these enzymes were purified and characterized [33,35]. Cellulose is nature's most abundant biopolymer, and long has been recognized as a potential source of sugars for biofuel production. Voget and colleagues [37] obtained several cellulolytic clones by functionally screening a soil metagenome library from which they purified and characterized a cellulase. Rees *et al*. [38] screened a lake water metagenome library and retrieved four cellulolytic clones. From a metagenome library representing the microbial community present in the rabbit's cecum, several clones with cellulose activities were revealed [39]. Functional screening of metagenome libraries from extreme (high salinity and alkalinity) environmental samples (soil from Soda Lake, California, and lake sediments from Africa and Egypt) also disclosed cellulolytic clones [19]. Cellulolytic enzymes isolated from environments with extreme temperatures and pH values are receiving a lot of interest as these enzymes are expected to be better adapted to the conditions of industrial processes, such as the decomposition of recalcitrant plant cell wall biomass into fermentable sugars.

Chitin, a compound of the fungal cell wall, is the second most abundant natural biopolymer that is broken down by chitinases. Cottrell *et al*. [40] acquired clones with chitinase activities from metagenome libraries derived from marine samples (filtrated from coastal sea water and estuarine water near the Delaware Bay). Hemicellulose consists primarily of xylan and constitutes the second most abundant polymer in plant biomass. Xylanase activities were detected and expressed from metagenome libraries representing the microbial communities of an insect gut [41], and the waste water from a dairy farm [42].

The ester linkage between the 4-O-methyl-D-glucuronic acid of glucuronoxylan and lignin alcohols is one type of covalent linkage connecting lignin and hemicellulose in plant cell walls. Esterases, which belong to the group of carboxylester hydrolases, hydrolyze such linkages. Esterase activities were detected from metagenome libraries of soil [43,44], lake water [38], drinking water [43], and the micro flora from bovine rumen [45]. From these libraries, clones with endo-β-1,4-glucanase activity and a clone with cyclodextrinase activity were identified [45]. Agarases are enzymes that liquefy agar by cleaving either the polymer's α-L-(1,3) linkage or its β-D-(1,4) linkage. Voget *et al*. [34] discovered six agarase genes in a soil metagenome library. The same library yielded two clones with pectate lyase activity, and one clone with 1,4-α-glucan branching enzyme activity [34]. Table 1 summarizes the enzymes discovered via function-based screening, their metagenome origin, and the library types and sizes.

**Table 1:** Recently identified plant biomass-degrading enzymes through metagenomic approaches (metagenome libraries screening for enzyme activity)

Enzyme name	Metagenome DNA source	Library vector	Insert size	Number of clones screened	Positive clones	Reference
Agarase	Soil from an unplanted field	Cosmid	25–40 kb	1,523	12 clones (belong to six genes)	[34]

Amylase	Environmental (US patent number 5,958,672)	Lambda		50,000	15 clones (belong to three enzymes)	[33]

Amylase	Soil from an unplanted field	Cosmid	25–40 kb	1,523	1 clone	[34]

Amylase	Soil from the junction of the groundwater table	Plasmid	2–7 kb	30,000	1 clone	[35]

Amylase	Soil and compost from the surface layer of a private garden	Plasmid	1.4–6.5 kb	31,967	38 clones	[36]

Cellulase	Various lake water samples from East Africa	Lambda	2–10 kb	114,000	4 clones	[38]

Cellulase	Soil from an unplanted field	Cosmid	25–40 kb	1,523	1 clone	[34,37]

Cellulase	Soda lake sediments from Wadi el Natrun, Egypt	Lambda	2.0–5.5 kb	35,000	1 clone	[19]

Cellulase	A soda lake (Wadi el Natrun, Egypt) alkaline microcrystalline cellulose medium enrichment	Lambda	2–6 kb	37,000	1 clone	[19]

Cellulase	Rabbit cecum contents	Cosmid	22–47 kb	32,500	11 clones (representing six genes)	[39]

Chitinase	Coastal seawater outside the Delaware Bay	Lambda	1.8–4.2 kb	75,000	2 clones	[40]

Chitinase	Estuarine water inside the Delaware Bay	Lambda	5.0–6.1 kb	75,000	9 clones	[40]

Cyclodextrinase	Bovine rumen micro flora	Lambda	Average 5.5 kb	14,000	1 clone	[45]

Endo-β-1,4- glucanase	Bovine rumen micro flora	Lambda	Average 5.5 kb	14,000	9 clones	[45]

Esterase	Various lake water samples from East Africa	Lambda	2–10 kb	130,000	2 clones	[38]

Esterase	Bovine rumen micro flora	Lambda	Average 5.5 kb	14,000	12 clones	[45]

Esterase	Crude oil springs contaminated soil	Cosmid	25–40 kb	2,500	1 clone	[43]

Esterase	Biofilms growing with a drinking water network	Cosmid	25–40 kb	1,600	1 clone	[43]

Esterase	Pools of various environmental soils	Fosmid	30–40 kb	60,000	1 clone	[44]

Pectate lyase	Soil from an unplanted field	Cosmid	25–40 kb	1,523	2 clones	[34]

Xylanase	Insect gut (insects collected from various locations)	Lambda	3–6 kb	1,000,000	4 clones	[41]

Xylanase	Manure waste water from a dairy farm	Lambda	4–10 kb	5,000,000	1 clone	[42]

1,4-α-glucan branching enzyme	Soil from an unplanted field	Cosmid	25–40 kb	1,523	1 clone	[34]

kb = kilobase

#### Metagenome sequencing (homology-based identification)

Sequence-based screening methods rely on known conserved sequences, and cannot uncover non-homologous enzymes. Therefore, the drawback of this 'closed approach' is its failure to detect fundamentally different 'new' genes. However, unlike function-based methods, it can disclose target genes, regardless of gene expression and protein folding in the host, and irrespective of the completeness of the target gene's sequence. The success of this approach rests on meeting several conditions:

(1) The more complex the community, the larger must be the sequencing effort. Here, the development of new sequencing technology, such as the next-generation 454-pyrosequencing, has changed the outcome. For instance, one of the first metagenome projects was the exploration of microbial communities in the drainage from acid mines [12], wherein only three bacterial and three archaeal lineages were detected. Nowadays, metagenome projects using new sequencing technologies not only generate greater total base pair reads but also have more even coverage of species within the community [17].

(2) While the metagenomic approach captures representative DNA samples from diverse organisms, many sequence reads remain unassembled due to the variety of sizes of the environmental genomes, and their abundance. Therefore, a shift in focus emerged, from complete metagenome sequencing to bulk sequencing of as many possible genes and/or functions. In this latter approach, where there is less need to assemble sequences into contigs, the limiting factor becomes the lengths of the fragments that can be obtained for high-throughput screening and cloning. Ideally, the fragments must be long enough to contain the full open reading frame for the functions of interest. Accordingly, optimized 454 sequencing (approximately 450 nucleotide (nt) sequence length) looks more promising than extremely high-volume short-run (25 nt) sequencing [46,47], but still has its limitations for downstream cloning and expression of genes like GHase that vary in length from less than 1 kb to more than 20 kb. Gene-finding tools, such as MetaGene, were demonstrated to predict 90% of shotgun sequences [48].

(3) New bioinformatics tools are needed for data mining, based not only on primary sequence homology but also able to predict protein structures, putative catalytic sites, and activities. With the betterment of protein classification tools, models might be designed to correlate enzyme mechanisms and protein folding. Based on this folding and the creation of putative active sites, gene function can be predicted [49-54]. We anticipate that soon sequence-based metagenome databases searches combined with bioinformatics tools will have a greater influence on mining novel biocatalyst genes than function-based methods.

Several publications describe searching metagenome sequence databases in prospecting for genes and their enzymes that will be useful in biofuel production. For example, in sequencing a metagenome library of hindgut microbiota from the largest family of wood-feeding termites (Termitidae), Warnecke and colleagues [55] generated 71 million base pairs of sequence data. By detecting complete domains using global alignment, they identified more than 700 domains homologous to glycoside-hydrolase catalytic corresponding to 45 different carbohydrate-active enzymes (CAZy) families [56], including a rich diversity of putative cellulases and hemicellulases. Schlüter and colleagues [57,58] sequenced, using 454-pyrosequencing technology, a metagenome library of the microbial community from the biogas fermenter of an agricultural biogas plant. From among the 141 million base pair sequences generated, bacteria that played dominant roles in methanogenesis and gene-encoding cellulolytic functions were identified from among the *Clostridia *spp. [57,58]. In the near future, we anticipate more publications on mining novel biocatalysts using sequence-based metagenome searches.

### A survey of available metagenome databases

According to GOLD [17], of the 137 metagenomic projects in the various stages of sequencing, 46 were finished (including 43 projects from 22 different environmental samples and 3 simulated communities), and the resulting data are available through the IMG/M website [18,56]. By searching through the list of 'genes with Pfam' (the protein family database) from every metagenome on the IMG/M website, our group retrieved 4,874 glycosyl/GHase homologues from these 46 completed metagenome databases. Then, to gain better insight into the diversity and representation of putative glycosyl hydrolases in these metagenomes, we downloaded the databases of translated sequences from all 43 environmental metagenome projects, and blast-searched them against the CAZy sequences for homologues of GHases (van der Lelie *et al*., unpublished data). As shown in Table 2, using an e value < 10^-40 ^as a cut-off threshold, we recognized 7,338 putative GHase homologues. The table also gives the metagenome size of each environmental sample, the number of homologues, and the number of putative GHases found per million base pairs. Generally, metagenome samples taken from environments that are characterized by a steady input and turnover of complex plant cell wall biomass have an increased abundance of putative GHases: the metagenomes from microbial communities derived from termite, human, and mouse guts displayed more putative GHase homologues (approximately 1.5% total gene count) than those from other samples, such as human oral microflora, uranium-contaminated groundwater or Singapore air sample (approximately 0.3% total gene count). Many of these metagenomic projects originally were targeted on different subjects, such as sulfate reduction, metal tolerance or marine archaeal anaerobic methane oxidation (denoted in descriptions of metagenome sources in Table 2). Table 3 lists the five most abundant GHase families for each environment (except the marine archaeal anaerobic methane-oxidation community that had only three GHase matches on 2.1 million base pairs). In most metagenomes, GHase family 13 represents the most abundant family. Its known activities include the following: α-amylase; pullulanase; cyclomaltodextrin glucanotransferase; cyclomaltodextrinase; trehalose-6-phosphate hydrolase; oligo-α-glucosidase; maltogenic amylase; neopullulanase; α-glucosidase; maltotetraose-forming α-amylase; isoamylase; glucodextranase; maltohexaose-forming α-amylase; maltotriose-forming α-amylase; branching enzyme; trehalose synthase; 4-α-glucanotransferase; maltopentaose-forming α-amylase; amylosucrase; sucrose phosphorylase; malto-oligosyltrehalose trehalohydrolase; isomaltulose synthase; and, amino acid transporter. The next most abundant is GHase family 23 (lysozyme type G; peptidoglycan lyase; also known in the literature as peptidoglycan lytic transglycosylase). Additionally, we found that members of the GHase family 2 (β-galactosidase; β-mannosidase; β-glucuronidase; mannosylglycoprotein endo-β-mannosidase; exo-β-glucosaminidase), and GHase family 3 (β-glucosidase; xylan 1,4-β-xylosidase; β-N-acetylhexosaminidase; glucan 1,3-β-glucosidase; glucan 1,4-β-glucosidase; exo-1,3-1,4-glucanase; α-L-arabinofuranosidase) are abundant in most environments. In fact, GHase family 13 (also known as the α-amylase family) is the largest sequence-based family of GHases, and encompasses several different enzyme activities and substrate specificities acting on α-glycosidic bonds. This might be a reason why GHase family 13 seemingly is the dominant family in most metagenomes. Clearly, homology, enzyme activity, and substrate specificity are not always well linked for GHases of the same family, thereby highlighting one weak point of homology-based screening for new GHase activities. Better classification and functional prediction of GHases should improve future bioprospecting of new ones for biofuel production.

**Table 2:** Glycosyl hydrolase homologues found in metagenome samples

Metagenome source^a^	Genome size (bp)	Gene count^b^	Glycosyl hydrolase matches^c^	Glycosyl hydrolase matches/total genes (%)^d^	Matches/million base pairs^d^
Marine archaeal anaerobic methane oxidation community (methane oxidation, sulfate reducer) [62]	2,116,255	2,332	3	0.13	1.42

Acid mine drainage (acidic, metal tolerance, pink biofilm) [12]	10,830,886	12,559	73	0.58	6.74

Human gut community (gut microbiome of human) [63]	36,304,498	46,503	705	1.52	19.42

Hypersaline mat (marine microbial communities) [64]	84,253,870	135,922	786	0.58	9.33

Lake Washington formaldehyde enrichment (^13^C-labeled formaldehyde; ^13^C-labeled DNA isolated by CsCl purification) [21]	57,622,063	89,729	397	0.44	6.89

Lake Washington formate enrichment (^13^C-labeled formate; ^13^C-labeled DNA isolated by CsCl purification) [21]	17,570,569	28,700	114	0.40	6.49

Lake Washington methane enrichment (^13^C-labeled methane; ^13^C-labeled DNA isolated by CsCl purification) [21]	52,164,993	81,076	428	0.53	8.20

Lake Washington methanol enrichment (^13^C-labeled methanol; ^13^C-labeled DNA isolated by CsCl purification) [21]	50,245,961	77,229	373	0.49	7.42

Lake Washington methylamine enrichment (^13^C-labeled methylamine; ^13^C-labeled DNA isolated by CsCl purification) [21]	37,225,208	54,340	285	0.52	7.66

Mouse gut community (lean mouse) [65]	6,511,633	8,510	119	1.40	18.27

Mouse gut community (obese mouse) [65]	4,200,364	5,382	58	1.08	13.81

*Olavius algarvensis *microbiome delta (sulfate reducer, symbiont) [66]	19,918,898	15,092	41	0.27	2.06

*Olavius algarvensis *microbiome gamma (sulfate reducer, symbiont) [66]	9,964,793	6,026	16	0.27	1.61

Singapore air sample [67]	75,598,288	91,635	514	0.56	6.80

Sludge Australian Phrap assembly (phosphate removal) [68]	53,048,954	30,590	177	0.58	3.34

Sludge US Jazz assembly (phosphate removal) [68]	41,128,538	16,840	126	0.75	3.06

Sludge US Phrap assembly (phosphate removal) [68]	56,608,360	34,254	260	0.76	4.59

Soil diversa silage (farm silage surface soil) [69]	152,406,385	184,374	1,078	0.58	7.07

TM7 (human oral microflora) [70]	3,451,819	3,908	14	0.36	4.06

Termite gut (cellulolytic, cellulose degrader, lignin degrader, symbiont) [55]	61,992,778	83,225	1,267	1.52	20.44

Uranium-contaminated groundwater (acidophile)	9,554,544	12,335	71	0.58	7.43

Whalefall sample (barophile) [69,71]	94,937,484	122,145	433	0.35	4.56

Total (or average for glycosyl hydrolase matches/total genes and matches/million base pairs)	937,657,141	1,142,706	7,338	0.64	7.83

^a^Data source: IMG/M [18]; ^b^only protein coding sequences were included (no RNA genes); ^c^translated sequences from all 43 environmental metagenome projects were blast-searched against the CAZy sequences for homologues of glycosyl hydrolases using an e value < 10^-40 ^as a cut-off threshold; ^d^Glycosyl hydrolase matches/total genes and matches per million base pairs provides an indication for the relative abundance of glycosyl hydrolases in the microbial community.

**Table 3:** Most abundant glycosyl hydrolase families found in different metagenome samples

Metagenome source^a^	Glycosyl hydrolase matches^b^	Most abundant glycosyl hydrolase family^c^
Marine archaeal anaerobic methane oxidation community (methane oxidation, sulfate reducer) [62]	3	GH16 (33%), GH2 (33%), GH38 (33%)

Acid mine drainage (acidic, metal tolerance, pink biofilm) [12]	73	GH13 (26%), GH15 (21%), GH57 (12%), GH28 (9%), GH18 (7%)

Human gut community (gut microbiome of human) [63]	705	GH13 (20%), GH3 (11%), GH2 (9%), GH1 (7%), GH31 (5%)

Hypersaline mat (marine microbial communities) [64]	786	GH13 (24%), GH2 (9%), GH3 (7%), GH65 (4%), GH57 (4%)

Lake Washington formaldehyde enrichment (^13^C-labeled formaldehyde; ^13^C-labeled DNA isolated by CsCl purification) [21]	397	GH13 (15%), GH23 (9%), GH3 (8%), GH2 (8%), GH94 (8%)

Lake Washington formate enrichment (^13^C-labeled formate; ^13^C-labeled DNA isolated by CsCl purification) [21]	114	GH13 (28%), GH23 (10%), GH2 (7%), GH94 (6%), GH28 (4%), GH8 (4%)

Lake Washington methane enrichment (^13^C-labeled methane; ^13^C-labeled DNA isolated by CsCl purification) [21]	428	GH13 (17%), GH94 (10%), GH23 (8%), GH3 (8%), GH57 (5%)

Lake Washington methanol enrichment (^13^C-labeled methanol; ^13^C-labeled DNA isolated by CsCl purification) [21]	373	GH13 (16%), GH23 (9%), GH3 (8%), GH94 (8%), GH2 (5%)

Lake Washington methylamine enrichment (^13^C-labeled methylamine; ^13^C-labeled DNA isolated by CsCl purification) [21]	285	GH23 (20%), GH13 (13%), GH57 (8%), GH17 (7%), GH3 (6%)

Mouse gut community (lean mouse) [65]	119	GH3 (10%), GH43 (9%), GH94 (9%), GH2 (8%), GH13 (8%)

Mouse gut community (obese mouse) [65]	58	GH68 (12%), GH13 (10%), GH43 (10%), GH94 (10%), GH2 (10%), GH3 (10%)

*Olavius algarvensis *microbiome delta (sulfate reducer, symbiont) [66]	41	GH23 (41%), GH13 (12%), GH57 (7%), GH2 (5%), GH3 (5%), GH5 (5%), GH28 (5%), GH43 (5%)

*Olavius algarvensis *microbiome gamma (sulfate reducer, symbiont) [66]	16	GH23 (31%), GH13 (19%), GH2 (6%), GH3 (6%), GH28 (6%), GH31 (6%), GH57 (6%), GH73 (6%), GH77 (6%), GH103 (6%)

Singapore air sample [67]	514	GH13 (13%), GH3 (9%), GH23 (9%), GH15 (8%), GH28 (5%)

Sludge Australian Phrap assembly (phosphate removal) [68]	177	GH13 (28%), GH23 (15%), GH16 (8%), GH103 (7%), GH3 (7%)

Sludge US Jazz assembly (phosphate removal) [68]	126	GH13 (20%), GH23 (12%), GH3 (9%), GH16 (7%), GH2 (6%)

Sludge US Phrap assembly (phosphate removal) [68]	260	GH13 (17%), GH23 (13%), GH3 (8%), GH16 (7%), GH94 (7%)

Soil diversa silage (farm silage surface soil) [69]	1,078	GH13 (22%), GH3 (9%), GH94 (8%), GH43 (5%), GH15 (5%), GH2 (5%)

TM7 (human oral microflora) [70]	14	GH13 (29%), GH1 (21%), GH57 (21%), GH4 (7%), GH25 (7%), GH 28 (7%), GH73 (7%)

Termite gut (cellulolytic cellulose degrader, lignin degrader, symbiont) [55]	1,267	GH13 (12%), GH94 (12%), GH5 (9%), GH3 (8%), GH2 (6%)

Uranium-contaminated groundwater (acidophile)	71	GH23 (18%), GH13 (10%), GH94 (7%), GH17 (6%), GH28 (6%), GH3 (6%)

Whalefall sample (barophile) [69,71]	433	GH23 (17%), GH13 (13%), GH3 (12%), GH2 (6%), GH103 (5%)

^a^Data source: IMG/M [18]; ^b^total number of GHase matches in each metagenome are given; translated sequences from all 43 environmental metagenome projects were blast-searched against the CAZy sequences for homologues of glycosyl hydrolases using an e value < 10^-40 ^as a cut-off threshold; ^c^the five most abundant glycosyl hydrolase families are listed. GHX is short for glycosyl hydrolase family X; percentages of each glycosyl hydrolase family are indicated inside parentheses.

### Future prospects

#### (i) Development of high through-put screening methods

Although the new ultra-fast sequencing technologies quickly generate a remarkable number of target gene candidates, functional assays are still needed to confirm them. Assays for protein function represent one of the most reliable and irreplaceable tools for mining target genes, and, therefore, developing high through-put functional screening methods is a priority for reducing the time exhausted in primary screening. Furthermore, such future screening methods might valuably be combined with other technologies, such as micro-arrays, biosensors, or proteomics tools to accelerate the discovery of new biocatalyst genes.

#### (ii) Advances in bioinformatics tools

The metagenomics approach provided valuable insight into a full range of microbial diversity in the environment, regardless of their cultivability. However, the complexity of microbial species, together with the limitations of the technology to cover fully whole genome sequences of every species present still pose a great challenge for metagenome research. A few bioinformatics programs are established for assembling and binning metagenome sequences, for gene prediction and annotation, estimating community composition, and data management (see Kunin *et al*. [60] for review). In addition, the European Union-funded 'MetaFunctions' project [61] also covers the development of 'metagenomes Mapserver', a data-mining system that correlates genetic patterns in genomes and metagenomes with contextual environmental data. Nevertheless, more innovative and sophisticated bioinformatics tools must be devised to assure continued valuable progress in the field of metagenomics.

## Conclusion

With the depletion of fossil fuels and growing environmental awareness, bioenergy production from renewable, non-food resources more and more enters into public focus. The natural gene diversity and complexity found in metagenomes is remarkable, affording us an ideal resource for mining of novel biocatalytics that efficiently break down recalcitrant plant biomass into fermentable sugars for generating biofuels and other chemical commodities. With the development of new biotechnologies and bioinformatics tools, our discovery of, and access to novel enzymes via metagenomic approaches potentially may significantly contribute to their future economical production from renewable resources.

## Abbreviations

AMD: acid mine drainage; CAZy: carbohydrate-active enzymes; GHase: glycosyl hydrolase; GOLD: Genomes OnLine Database; IMG/M: Integrated Microbial Genomes with Microbiome Samples; kb: kilobase; nt: nucleotide.

## Competing interests

The authors declare that they have no competing interests.

## Authors' contributions

LLL participated in the metagenome data analysis and wrote the manuscript. SRM wrote the scripts and run the metagenome BLAST analysis. SM participated in the data analysis and commented on the manuscript. ST provided input and commented on the manuscript. DvdL participated in data analysis, set the outlines for the manuscript, and critically reviewed and commented on the manuscript.
